# A household-based survey of knowledge, attitudes and practices towards dengue fever among local urban communities in Taiz Governorate, Yemen

**DOI:** 10.1186/s12879-016-1895-2

**Published:** 2016-10-07

**Authors:** Thaker A. A. Alyousefi, Rashad Abdul-Ghani, Mohammed A. K. Mahdy, Samira M. A. Al-Eryani, Abdulsalam M. Al-Mekhlafi, Yahia A. Raja, Shamusul Azhar Shah, John C. Beier

**Affiliations:** 1 Department of Hematology, Faculty of Medical Sciences, Al-Razi University, Sana’a, Yemen; 2 Department of Parasitology, Faculty of Medicine and Health Sciences, Sana’a University, Sana’a, Yemen; 3 Tropical Disease Research Center, University of Science and Technology, Sana’a, Yemen; 4 Faculty of Public Health and Informatics, Umm Al-Qura University, Mecca, Saudi Arabia; 5 Department of Community Health, Universiti Kebangsaan Malaysia Medical Centre, Kuala Lumpur, Malaysia; 6 Department of Public Health Sciences, University of Miami Miller School of Medicine, Miami, FL USA

**Keywords:** Dengue fever, Knowledge, Attitude, Practice, Taiz, Yemen

## Abstract

**Background:**

Yemen has witnessed several dengue fever outbreaks coincident with the social unrest and war in the country. The aim of the present study was to describe the knowledge, attitudes and practices (KAPs) of at-risk urban populations residing in Taiz, southwest of Yemen. In addition, factors possibly associated with poor preventive practices were investigated.

**Methods:**

A household-based, cross-sectional survey was conducted in three urban districts encompassing 383 households. Data on the socio-demographic characteristics and KAPs of the participating household heads were collected using a pre-designed, structured questionnaire. The association of socio-demographic characteristics, knowledge and attitudes of the population with poor preventive practices against dengue fever was then analyzed using logistic regression.

**Results:**

More than 90.0 % of respondent household heads had correct knowledge about fever, headache and joint pain as common signs and symptoms of dengue fever. Moreover, muscular pain and bleeding were perceived by more than 80.0 % of the respondents as being associated with dengue fever; however, only 65.0 % of the respondents reported skin rash as a sign of dengue fever. More than 95.0 % of respondents agreed about the seriousness and possible transmission of dengue fever; however, negative attitudes regarding the facts of being at risk of the disease and that the infection is preventable were expressed by 15.0 % of respondents. Despite the good level of knowledge and attitudes of the respondent population, poor preventive practices were common. Bivariate analysis identified poor knowledge of dengue signs and symptoms (OR = 2.1, 95 % CI = 1.24–3.68; *P =* 0.005) and its vector (OR = 2.1, 95 % CI = 1.14–3.84; *P =* 0.016) as factors significantly associated with poor preventive practices. However, multivariable analysis showed that poor knowledge of the vector is an independent predictor of poor preventive practices of the population (adjusted OR = 2.1, 95 % CI = 1.14–3.84; *P* = 0.018).

**Conclusion:**

The majority of people in urban communities of Taiz have a clear understanding of most signs/symptoms of dengue fever as well as positive attitudes towards the seriousness and possible transmissibility of dengue fever. However, negative attitudes regarding their perception of the risk and possible prevention of the infection are prevailing among a small proportion of the population and need to be targeted by educational campaigns. It appears that the good level of the population knowledge of the signs/symptoms of dengue fever and the factors contributing to the spread and control of its vectors did not translate into good practices.

## Background

Dengue fever is a systemic arboviral disease caused by the dengue virus and transmitted by infected female *Aedes* mosquitoes, mainly *Ae. aegypti* (primary vector) [1]. Infections can also be transmitted through blood transfusion, organ transplantation and possibly vertically from mother to child [2–6]. Although infection with dengue virus may be asymptomatic [7, 8], it may lead to a wide spectrum disease that ranges from non-severe fever to potentially fatal clinical manifestations [1]. Globally, 294 million inapparent and 96 million apparent dengue infections were estimated in 2010 [7]. Dengue virus has five serotypes [9], and infection with a certain serotype usually confers a lifelong serotype-specific immunity, but a temporary immunity to other serotypes [10, 11]. Moreover, more frequent and severe complications can occur in subsequent infection with a different serotype [10, 11]. Due to the absence of effective antiviral agents [1, 12], efforts focus on interrupting human-vector contact through targeting the adult vector and its immature stages by eliminating its breeding habitats in and near households [1]. Nevertheless, the incidence of dengue fever epidemics is escalating, and its endemic transmission expands to a wider geographical range [7]. It is noteworthy that the recombinant live-attenuated tetravalent dengue vaccine (CYD-TDV), commercially known as Dengvaxia®, was the first licensed vaccine against dengue, given as three doses at 0, 6 and 12 months [13]; however, its pooled efficacy over 25 months from the first dose was reported to be 65.6 % based on data derived from Phase 3 clinical trials from endemic countries in Asia and Latin America, with varying degrees of protection according to the virus serotype, age, disease severity and serostatus at vaccination [14]. Therefore, the Strategic Advisory Group of Experts on Immunization recommends the introduction of CYD-TDV only in high-endemicity settings, after careful assessment, where seroprevalence rates of the virus are 70 % or greater in the age group to be targeted by vaccination [15].

Dengue fever is a preventable infection, and success of dengue control depends largely on good knowledge, attitudes and practices (KAPs) of targeted communities towards the disease and its preventive measures. Community involvement after educational campaigns could be an effective approach to the prevention and control of dengue. In line with this view, it has been shown that community education could be more effective than insecticide spraying alone in reducing mosquito breeding habitats [16].

In 2003, AlHoot [17] reported IgG and IgM seronegativity for dengue fever among febrile and apparently healthy individuals from different localities in Yemen. Despite the increasing outbreaks of dengue fever in Yemen, very few studies have been published to document these outbreaks. Dengue fever outbreaks caused by dengue virus serotype 3 were reported in Al-Mukalla city, east of Yemen [18, 19]. Moreover, a case of imported dengue virus serotype 3 was also reported from an Italian man returning from a locality near Al-Mukalla in 2010 [20]. In Hodeidah, west of Yemen, 29.0 % (116/400) of hospitalized patients with fever and, at least, two signs or symptoms of dengue or dengue-like diseases were reported to be infected with dengue fever virus, predominantly of serotype 2 [21]. There is a lack of KAP studies on dengue fever among Yemeni people. Saied et al. [22] concluded that rural populations in Hodeidah governorate have an unclear understanding of dengue fever transmission, negative attitudes towards several features of the disease and poor practices against it.

The present social unrest and war in Yemen, particularly in Taiz, contribute to the incidence of dengue fever outbreaks (Ministry of Health and Population, unpublished data). Moreover, there is a need for documented proof on the KAPs of local populations in Taiz on the infection and its prevention. Therefore, the present study aimed at describing the KAPs of local urban communities in Taiz city towards dengue fever.

## Methods

### Study area and ethical clearance

**Fig. 1:**
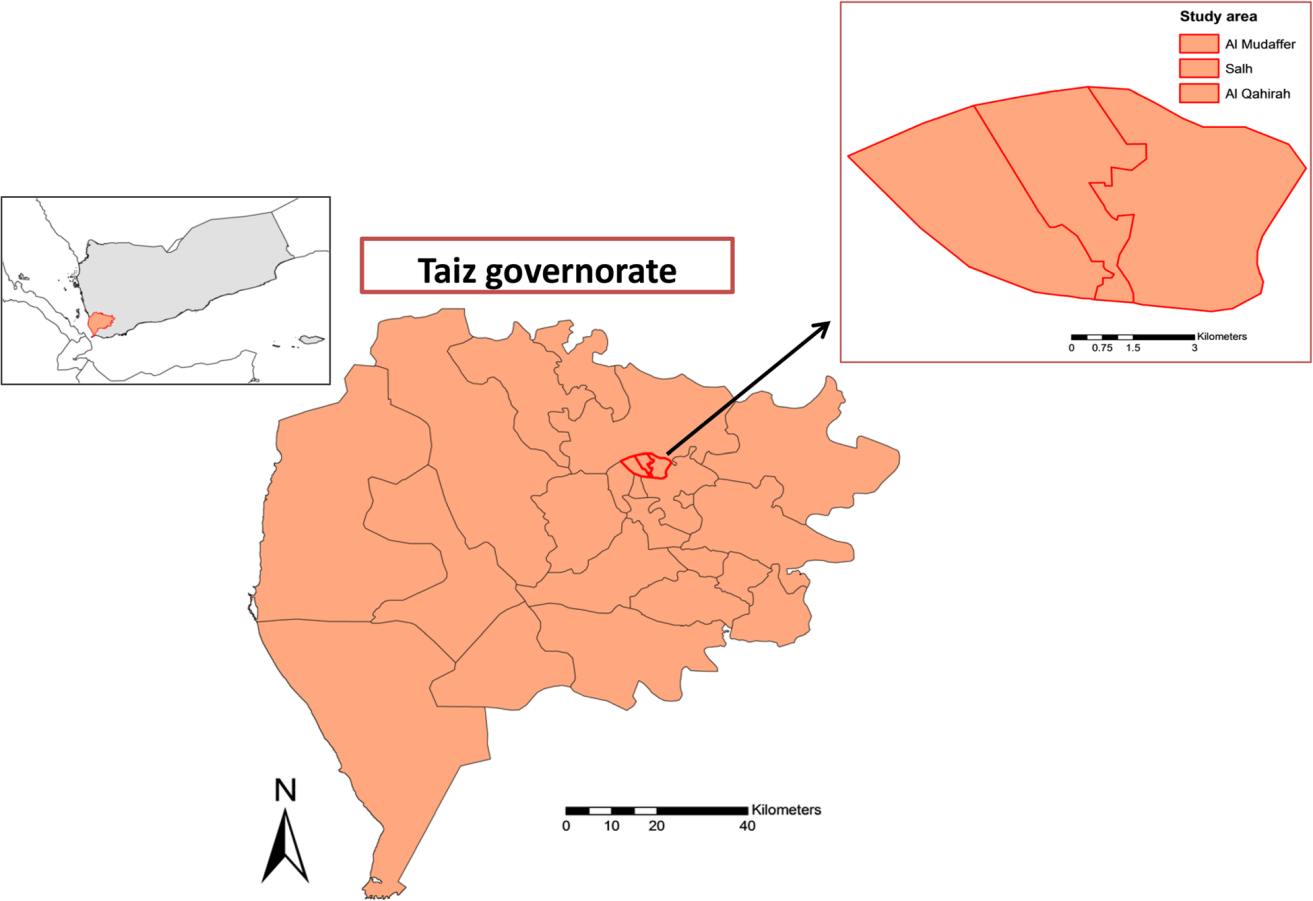
Map of Yemen and Taiz governorate showing the study area

This community-based, cross-sectional KAP study was conducted in the urban area of Taiz, a hinterland governorate in the southwest of Yemen, in the period from August to October 2015. Tiaz is located at the geographical coordinates of 13°34′44″N 44°01′19″E at an altitude of about 1400 m above the Red Sea level (Fig. 1). It is the most populous governorate in the country, with a total population of more than 3 million people. Of them, about 684,000 people live in urban areas [23].

The study protocol was approved by the Ethics Committee of the Faculty of Medicine and Health Sciences, University of Science and Technology, Sana’a, Yemen. Participation was on a voluntary basis, and informed consent was obtained from participants after explanation of the study objectives.

### Sample size and sampling strategy

According to the latest census, the total number of households in the urban areas of Taiz is 113,000 households [23]. The present study targeted 71,303 households in three urban dengue-endemic districts; namely, Al Qahirah, Al Mudhaffar and Salh. The sample size was calculated using Epi Info™ version 7.1.3 (Centers for Disease Control, Atlanta, US), using the following parameters: population size of 71,303 households, 5 % confidence limits and 95 % confidence level. The expected frequency of the outcome was considered 50 % because the study covered several potential variables. Accordingly, the minimum sample size calculated was 382 households. Households were randomly selected and household heads were invited to participate in the study after obtaining their informed consent. If the household head was not present or refused to participate, the head of the next household was included until reaching the sample size required.

Data were collected using a structured questionnaire through face-to-face interviews. Interviewers were trained before conducting the survey to ensure that the questionnaires were well understood by the surveyors, avoiding the difference in the definitions and interpretations of concepts used. The questionnaire included closed-ended questions about socio-demographic data, knowledge of the symptoms, transmission and vector of dengue fever, attitudes and practices of the respondents towards dengue fever.

### Statistical analysis

Data were verified and analyzed using the IBM SPSS Statistics version 21.0 for Windows (IBM Corp., Armonk, NY, USA). Variables were presented as proportions, and the differences were tested using Pearson’s chi-square test. To identify the predictors of poor practices, independent and dependent variables were converted into scores and categorized as poor and good. Scores of “one” and “zero” were given to the correct and incorrect knowledge or practices, respectively. For attitudes, the answers “disagree”, “not sure”, “agree” and “strongly agree” were given the scores of 1, 2, 3 and 4, respectively. Knowledge and practices were considered poor if the score was lower than or equal to the half of the total score (3, 2.5 and 4 for knowledge of symptoms, transmission and mosquito, respectively, and 4 for practices). Similarly, attitudes were considered negative if the score was lower than or equal to the half of the total score, which was 4.5. The associations between independent and dependent variables were tested using Pearson’s chi-square test. The odds ratio (OR) and its 95 % confidence interval (CI) were also reported. Variables with *P*-values less than or equal to 0.2 were further analyzed by multivariable analysis using a forward conditional stepwise logistic regression model.

## Results

### Socio-demographic characteristics of the study respondents

Respondent distribution according to socio-demographic characteristics is shown in Table 1. Of the respondents, 48.6 % were females, 18.0 % were illiterate and 37.5 % had a paid job.

**Table 1:** Socio-demographic characteristics of the study respondents (*N* = 383)^a^

Variable	Respondent distribution *n* (%)
Age (years)	
< 30	142 (40.1)
40–30	127 (35.9)
41–50	50 (14.1)
51–60	26 (7.3)
> 60	9 (2.5)
Sex	
Male	197 (51.4)
Female	186 (48.6)
Educational level	
Illiterate	65 (18.0)
Primary school	56 (15.5)
Secondary school	96 (26.6)
University	144 (39.9)
Paid job	
Yes	75 (37.5)
No	125 (62.5)

^a^31, 22 and 183 of respondents did not indicate their age, education and job, respectively

### Correct knowledge of respondent household heads on dengue fever

Table 2 summarizes the correct knowledge of household heads on dengue fever signs and symptoms, its transmission and the practices that can contribute to the spread of its vector mosquitoes. The majority of the 383 respondents correctly perceived that fever (98.7 %), headache (94.8 %) and joint pain (95.8 %) are main signs and symptoms of dengue fever. In addition, more than 80.0 % of respondents correctly identified pain behind the eyes, muscular pain and bleeding as signs and symptoms of dengue fever. However, skin rash was the least frequent symptom of the disease correctly identified by the respondents, being recognized by 65.0 % of them.

**Table 2:** Correct knowledge of household heads about dengue fever in urban communities of Taiz governorate, Yemen (*N* = 383)

Knowledge items	Correct Knowledge	
	n	% (95 % CI)
Dengue fever signs and symptoms		
Fever	378	98.7 (97–99)
Headache	363	94.8 (92–97)
Joint pain	367	95.8 (93–97)
Muscle pain	314	82.0 (78–86)
Eye pain	337	88.0 (84–91)
Skin rash	249	65.0 (60–70)
Bleeding	309	80.7 (76–84)
Dengue fever transmission		
Flies do not transmit dengue	309	80.7 (76–84)
Contact with infected patients does not transmit dengue	326	85.1 (81–88)
Drinking contaminated water does not transmit dengue	264	68.9 (64–73)
Eating contaminated food does not transmit dengue	298	77.8 (73–82)
Dengue is transmitted by blood transfusion	292	76.2 (72–80)
Dengue is transmitted by black mosquitoes^a^	324	84.6 (81–88)
Mosquitoes bite at daytime	229	70.7 (66–75)
Factors mentioned to increase mosquito spread		
Stagnant water	312	96.3 (94–98)
Keeping water containers opened	296	91.4 (88–94)
Factors mentioned to reduce mosquito spread		
Using mosquito nets	300	92.6 (89–95)
Using window screens	311	96.0 (93–98)
Insecticide spraying	295	91.0 (87–94)
Covering water containers	299	92.3 (89–95)
Drying stagnant water	292	90.1 (86–93)
Using repellent creams	227	70.1 (65–75)
Smoldering	250	77.2 (72–81)
Rubbish disposal	309	95.4 (93–97)
Cutting trees near houses	282	87.0 (83–90)

^a^Sample size is 324 for the rest of questions

Regarding the recognized mode of dengue transmission, 84.6 % of respondents knew that the black mosquito is the vector transmitting dengue fever. Of whom, only 70.7 % knew that these mosquitoes are day-biters. In addition, about three-quarters of the respondents knew the possibility of dengue fever transmission via blood transfusion. On the other hand, comparable proportions of respondents, ranging between 68.1 and 85.1 %, had correct conceptions that flies, contact with infected people, eating contaminated food or drinking contaminated water have no role in the transmission of dengue fever (Table 2).

Stagnant water and keeping water in uncovered containers were recognized by more than 90.0 % of respondents as factors contributing to the spread of dengue-transmitting vector mosquitoes. In addition, more than 90.0 % of respondents knew that using mosquito nets, window screening, covering water containers, drying stagnant water and rubbish disposal are preventive measures that could contribute to reducing the spread of vector mosquitoes. However, cutting trees near houses, smoldering and using repellent creams as preventive measures that could reduce the spread of vector mosquitoes were recognized by 87.0, 77.2 and 70.1 % of respondents, respectively.

### Attitudes of respondent household heads towards dengue fever

Table 3 shows the positive attitudes of respondent household heads towards dengue fever. The majority of respondents agreed about the seriousness of dengue fever and its transmissibility, 97.7 and 96.3 %, respectively. Lower positive attitude rates of 75.5 and 84.6 % were expressed regarding the facts of being at risk of contracting dengue fever and that the infection can be prevented, respectively.

**Table 3:** Positive attitudes of household heads towards dengue fever in the urban communities of Taiz governorate, Yemen (*N* = 383)

Attitude items	Positive attitude	
	n	% (95 % CI)
Dengue fever is a serious disease	374	97.7 (96–99)
Dengue is a transmissible disease	369	96.3 (94–98)
I am at risk of dengue fever	289	75.5 (71–79)
Dengue fever can be prevented	324	84.6 (81–88)

*CI* confidence interval

### Good practices of respondent household heads towards dengue fever

Table 4 summarizes good preventive practices against dengue-transmitting mosquitoes among respondent household heads. Covering water containers was the most common good practice among 94.8 % of respondent household heads; followed by window screening (77.5 %), drying water collections around houses (72.1 %) and owning mosquito nets (65.0 %). However, about a half of the respondents did not follow good practices of insecticide spraying or sleeping under mosquito nets. Using creams and fans for repelling mosquitoes were the least frequent practices mentioned by the respondent household heads, being reported by 31.3 and 16.2 % of respondents, respectively.

**Table 4:** Good preventive practices of household heads against dengue fever in the urban communities of Taiz governorate, Yemen (*N* = 383)

Practice items	Good practice	
	n	% (95 % CI)
Insecticide spraying	186	48.6 (44–54)
Having mosquito nets	249	65.0 (60–70)
Sleeping under mosquito nets	183	47.8 (43–53)
Using fans for repelling mosquitoes	62	16.2 (13–20)
Window screening	297	77.5 (73–81)
Drying water collections around houses	276	72.1 (67–76)
Covering water containers	363	94.8 (92–97)
Using creams for repelling mosquitoes	120	31.3 (27–36)

*CI* confidence interval

### Analysis of socio-demographic factors, knowledge and attitudes associated with poor practices

Factors possibly associated with poor preventive practices were analyzed by bivariate and multivariable analyses (Table 5). Using bivariate analysis, poor knowledge of dengue signs and symptoms (OR = 2.1, 95 % CI = 1.24–3.68; *P =* 0.005) and its vector (OR = 2.1, 95 % CI = 1.14–3.84; *P =* 0.016) were significantly associated with poor preventive practices among respondents. However, multivariable analysis showed that poor knowledge of dengue vector (adjusted OR = 2.1, 95 % CI = 1.14–3.84; *P* = 0.018) was an independent factor associated with poor preventive practices among respondents (Table 5).

**Table 5:** Analysis of socio-demographic factors, knowledge and attitudes associated with poor practices

Variable	Poor practices			
	N	n (%)	OR (95 % CI)	*P* value
Age (years)				
> 50	35	15 (42.9)	Reference	
30-50	177	90 (50.8)	1.4 (0.66–2.87)	0.389
< 30	142	61 (43.0)	1.0 (0.48–2.12)	0.991
Sex				
Female	186	93 (50.0)	Reference	
Male	197	88 (44.7)	0.8 (0.54–1.21)	0.296
Education				
University	144	64 (44.4)	Reference	
Secondary school	96	44 (45.8)	1.0 (0.59–1.82)	0.908
Primary school	56	30 (53.6)	1.2 (0.58–2.32)	0.667
Uneducated	65	35 (53.8)	1.3 (0.67–2.54)	0.436
Paid job				
Yes	75	30 (40.0)	Reference	
No	125	47 (37.6)	0.9 (0.50–1.63)	0.736
Knowledge of signs and symptoms				
Good	316	139 (44.0)	Reference	
Poor	67	42 (62.7)	2.1 (1.24–3.68)	0.005
Knowledge of transmission				
Good	323	158 (48.9)	Reference	
Poor	60	23 (38.3)	0.7 (0.37–1.14)	0.132
Knowledge of vector*				
Good	272	118 (43.4)	Reference	
Poor	52	32 (61.5)	2.1 (1.14–3.84)	0.016
Attitudes				
Positive	339	157 (46.3)	Reference	
Negative	44	24 (54.5)	1.4 (0.74–2.61)	0.303

Scores ≤ the half of total scores were considered poor or negative; *identified as an independent predictor of poor practices using multivariable analysis (Adjusted OR = 2.1, 95 % CI = 1.14–3.84; *P* = 0.018)

## Discussion

Globally, dengue fever is the most common vector-borne viral infection in the current century [24]. It tends to be of urban and peri-urban distribution, though it occurs in rural areas [25]. With the increasing incidence of dengue outbreaks in Yemen, the present study describes the KAPs of at-risk populations pertaining to dengue fever in three urban districts of Taiz. KAP surveys are of utmost importance in determining effective evidence-based prevention and control strategies through changing poor KAPs. Up to the best of our knowledge, this is the first study on the KAPs of Yemeni people in urban areas towards dengue.

In the present study, most urban community respondents were able to correctly identify fever, headache, joint pain, muscle pain, pain behind the eyes and bleeding as prominent signs and symptoms of dengue fever. However, skin rash was the least frequent symptom correctly recognized by the respondents. This is in agreement with a recent study on the KAPs of rural communities that reported the awareness of more than 90.0 % of respondents of the dengue fever symptoms [22]. Fever was the most frequently identified clinical presentation by the respondents, and this finding is consistent with previous studies from different countries [22, 26–29]. Because fever can be a sign of several febrile diseases endemic in Yemen such as malaria, people have to be educated about some other specific signs not sufficiently perceived by local populations such as rash and bleeding. Raising awareness about these signs and symptoms could help them distinguish dengue fever from other febrile infectious diseases, taking into consideration that only about two-thirds were able to correctly identify rash as a symptom of dengue.

In comparison to the recognition of dengue fever signs and symptoms, the perceived knowledge of the respondents about transmission of dengue fever by the black mosquito, a term used locally for describing the *Aedes* mosquitoes, was relatively lower (82.2 %). The lower level of knowledge among urban populations of Taiz that mosquitoes are vectors of dengue fever is comparable to that (83.4 %; 671/804) recently reported by Saied et al. [22] among Yemeni rural populations. In contrast, higher levels of knowledge were reported from northern Thailand (98.0 %), Nepal (92.0 %) and Pakistan (86.9 %) [28, 30, 31]. Of the respondents reporting that black mosquitoes transmit dengue fever, about two-thirds realized that these mosquitoes mainly transmit dengue fever during the daytime. This finding is higher than that recently reported among rural communities in Hodeidah, where about a third of the respondents perceived the daytime transmission of dengue fever [22]. In fact, malaria prevalence in the study areas could contribute to mistaken beliefs about the transmission of dengue fever by the same vector *Anopheles* mosquito. Therefore, differences in the characteristics, biting behaviors and habitat between malaria and dengue vector mosquitoes should be considered when tailoring educational campaigns to local communities about the prevention and control of dengue fever. Blood transfusion has been recently recognized as a possible mode of dengue fever transmission [4, 32, 33]; however, it was correctly identified by about three-quarters of the respondents as a source of dengue fever transmission.

Despite the low illiteracy rate (18.0 %) of the respondents in the present study, about 20 % of household heads have misconceptions about the modes of dengue fever transmission, including flies, contact with infected people, drinking contaminated water or eating contaminated food. In a recent study among rural populations in Hodeidah governorate, Saied et al. [22] reported that about 52.2 % (420/804) of the study population believe that dengue can be transmitted through contact with infected people. Such misconceptions may affect the practices of local populations for the prevention and control of the disease, which could be either poor or insufficient. Therefore, correction of mistakenly perceived modes of transmission should be considered to guide the health authorities for adapting forthcoming interventions for promoting best practices among populations of endemic areas. These misconceptions about the transmission modes have also been reported from other countries. In Jamaica, 33.5 and 28.2 % out of 188 parents of children attending child health clinics in an endemic area believe that dengue fever can be transmitted by flies and ticks, respectively [27]. Similarly, a recent KAP study in Nepal reported that 32.0, 42.0, 51.0 and 56.0 % of 589 participants in a cross-sectional survey in central Nepal believe that dengue fever can be transmitted by flies, by ticks, through food and water or by direct contact, respectively [31]. These mistaken beliefs about the modes of transmission may account for the negative attitude of 15.4 % of the respondents that dengue fever is not preventable.

The present study shows that the majority of the respondents (>90.0 %) recognized the role of stagnant water and keeping water in uncovered water containers as factors contributing to the spread of vector mosquitoes. This finding is comparable to that reported in a study in Nepal, where more than 90.0 % of lowland and highland populations identified stagnant water collections around houses as the breeding sites of mosquitoes [31]. Moreover, more than 90.0 % of the respondents were aware of the role of using mosquito nets, window screening, covering water containers, drying stagnant water and rubbish disposal as preventive measures that could contribute to reducing the spread of vector mosquitoes. However, fewer rates of perception were found regarding the role of cutting trees near houses, evaporation and smoldering and using repellent creams as preventive measures contributing to the reduction of dengue-transmitting mosquitoes. Apart from the good knowledge of people about the factors contributing to the spread of vector mosquitoes and the preventive measures to their reduction, the intriguing issue is the translation of such knowledge into practice, which was not evident among the respondents in the present study. Therefore, it is vital to seal the existing gap between knowledge and practices after gaining a better understanding of the reasons for not translating perceived knowledge into preventive practices.

The agreement that dengue fever is dangerous and can be transmitted among the population by the respondents of the present study is consistent with previous studies from other Asian countries [31, 34], where good attitudes towards the seriousness and/or transmissibility nature of dengue fever were found. In the present study, the negative attitudes among about 15.0 % of respondents from urban communities as regards not being exposed to the risk of dengue fever and that dengue fever is preventable are in line with the negative attitudes recently reported among the rural communities towards the prevention of dengue fever in Hodeidah governorate [22]. It is noteworthy that differences among various studies in relation to the attitudes of surveyed communities could be attributed to several factors, including the socio-economic status, educational levels and cultural aspects. Therefore, the success of future educational campaigns in fixing the gaps imposed by such negative attitudes of local populations pertaining to the risk and possible prevention of dengue could largely contribute to the control of the disease.

With the exception of the good practices of covering water containers, window screening and drying water collections around houses, other preventive practices are still not adopted by about a half of respondents. It is noteworthy that socio-demographic factors including age, educational level and paid job status were not predictors of poor practices in the present study. This finding could be attributed to the high literacy rate among urban residents and that the responses were obtained from the household heads. This is in contrast to the finding by Saied et al. [22], where a low educational level was a predictor of poor practices against dengue fever among rural populations in Yemen.

In the present study, a strong link exists between poor knowledge of respondents and their poor preventive practices as shown by bivariate analysis and confirmed by multivariable analysis. Poor knowledge of the vector mosquitoes was the independent factor significantly associated with poor practices of the respondents. This finding is consistent with that recently reported by Saied et al. [22] who found an association between knowledge and practices pertaining to the prevention of dengue fever among rural populations in Hodeidah. Similar associations between knowledge and practices have been recently reported from northern Thailand [28], Nepal [31] and Laos [35]. However, the association between knowledge and practices in the present study is in line with those from Jamaica [27], rural Malaysia [34] and Thailand [36], where good knowledge did not translate into good practices to reduce vector populations.

Despite being the first to uncover the KAPs of urban communities of Taiz towards dengue, the findings of the present survey have to be cautiously interpreted for several considerations. The small sample size may limit the precision of certain bivariate relationships between variables. Therefore, comparison across certain risk factors might not have enough sample or power. In addition, the present community survey adopted a 50.0 % cut-off score to divide good and poor knowledge because there was no similar knowledge questionnaires used previously. The questionnaires were mainly about general prevention knowledge but certainly could not cover the various and specific community correctness score. This might have a potential limitation when interpreting certain knowledge items. In other words, it is not necessary that one answer should be correct for all participants. For instance, bed nets may be valuable for infants but not working-age adults in reducing the risk of dengue infection. It is noteworthy that in spite of such limitations, the present findings unveil the existing gaps in the KAPs of urban communities in endemic areas of Taiz and highlight the need for further large-scale studies that consider the possible limitations for the sake of a better generalizability of results.

## Conclusions

In conclusion, the majority of people in urban communities of Taiz have a clear understanding of fever, headache, joint pain and pain behind the eyes as common signs and symptoms of dengue fever. However, a considerable proportion exhibits vague perception of other signs and symptoms, including muscle pain, skin rash and bleeding as being associated with dengue fever. Although the majority of people in urban communities of Taiz show positive attitudes towards the seriousness and possible transmissibility of dengue fever, negative attitudes regarding their perception of the risk and possible prevention of the infection are prevailing among a small proportion of urban communities and need to be targeted by educational campaigns. It appears that the good level of the population knowledge of the signs/symptoms of dengue fever and the factors contributing to the spread and control of its vectors did not translate into good practices. Large-scale educational campaigns for changing poor KAPs of populations in endemic areas should be launched to augment the negligible efforts to control dengue fever. In addition, there is a need for conducting future intervention studies to address the existing gaps in the knowledge and practices related to the prevention of dengue fever.

## Abbreviations

CI: confidence interval
CYD-TDV: recombinant live-attenuated tetravalent dengue vaccine
KAP: knowledge, attitudes and practices
OR: odds ratio
SPSS: Statistical Package for the Social Sciences
